# Osteoarthritis and other long-term health conditions in former elite cricketers

**DOI:** 10.1016/j.jsams.2017.10.013

**Published:** 2018-06

**Authors:** Mary E. Jones, Madeleine A.M. Davies, Kirsten M. Leyland, Antonella Delmestri, Angus Porter, Jason Ratcliffe, Nick Peirce, Julia L. Newton, Nigel K. Arden

**Affiliations:** a University of Oxford, Botnar Research Centre, UK; b Nuffield Department of Orthopaedics, Rheumatology, and Musculoskeletal Sciences, UK; c Professional Cricketers’ Association, The Laker Stand, Kia Oval, UK; d England and Wales Cricket Board, National Cricket Performance Centre, UK

**Keywords:** Heart diseases, Mental health, Prevalence, Aging, Sports, Athletes

## Abstract

**Objectives:**

This study aimed to describe the prevalence and risk of chronic conditions in former elite cricketers compared to a normal population, and describe wellbeing in former elite cricketers.

**Design:**

Cross-sectional study.

**Methods:**

Former elite cricketers, recruited from the Professional Cricketers’ Association, completed a self-report cross-sectional questionnaire. The English Longitudinal Study of Ageing (ELSA) served as the normal population. The prevalence of self-reported, GP-diagnosed conditions (heart problems, hypertension, stroke, diabetes, asthma, dementia, osteoarthritis (OA), total hip replacement (THR), total knee replacement (TKR), anxiety, depression) were reported for both population samples. Standardised morbidity ratios (SMRs) compared chronic conditions in sex-, age- and BMI-matched former cricketers (n = 113) and normal population (n = 4496).

**Results:**

Heart problems were reported by 13.3% of former cricketers, significantly lower than the normal population, SMR 0.55 (0.33–0.91). Former cricketers reported 31.9% hypertension, 1.8% stroke, 6.2% diabetes, 15.0% asthma, and no dementia, none significantly different to the normal population. OA, THR, and TKR were reported by 51.3%, 14.7% and 10.7% of former cricketers, respectively, significantly higher than the normal population, SMRs 3.64 (2.81–4.71), 3.99 (2.21–7.20) and 3.84 (1.92–7.68). Anxiety and depression were reported by 12.4% and 8.8% of former cricketers, respectively, SMRs 3.95 (2.34–6.67) and 2.22 (1.20–4.14). 97% of former cricketers reflected they would undertake their cricket career again, 98% agreed that cricket enriched their lives.

**Conclusions:**

Heart problems were significantly lower, while OA, THR, TKR, anxiety, and depression were significantly higher in the former cricketers compared to the normal population (ELSA). Most former cricketers reflected positively on their career.

## 1 Introduction

Cricket is a popular sport worldwide at both the recreational and elite levels, with an estimated 1 million players in the UK and 1.3 million players in Australia.^1^, ^2^ Physical activity has a wide number of health benefits, so it is important to encourage a sport such as cricket at all levels. However, understanding the possible negative outcomes of long-term sport participation may help in prevention and mitigation of these risks and contribute to informed participation.

Few investigations have considered the long-term health (i.e. heart problems, hypertension, stroke, diabetes, asthma, dementia, osteoarthritis (OA), total hip replacement (THR), total knee replacement (TKR), anxiety, and depression) of former elite athletes. To the best of our knowledge no studies have been conducted in former elite cricketers with a holistic view of physical and mental health, and overall wellbeing; such a study would provide indications of overall health benefits from this popular sport.^3^, ^4^

Physical activity, especially sport and recreational activities, has been shown to have major benefits in primary and secondary prevention of chronic conditions that are leading causes of death and disability, such as chronic heart disease (CHD) and hypertension,^5^, ^6^, ^7^ and to be associated with decreased incidence of stroke and diabetes.^8^ Research suggests an inverse relationship between regular physical activity in midlife and older age and incidence of dementia in healthy adults.^9^, ^10^ Elite athletes would be expected, therefore, to benefit from these positive effects of physical activity. Concurrently, OA has been suggested to be more common in certain former elite athletes, such as footballers, perhaps due to injury rates.^4^, ^11^, ^12^ Studies of current elite athletes have found comparable rates of mental health conditions as a general population sample, though athletes were not followed up after their transition out of elite sport, when many athletes experience depression and anxiety.^13^, ^14^, ^15^

While clinical diagnoses are important outcome measures, how athletes reflect on their sporting career may indicate how their career affected their wellbeing. Therefore, the career reflection of participants was investigated as an indication of wellbeing.

The aim of this study is primarily to describe the prevalence and risk of chronic conditions in former elite cricketers compared to a normal population, and to describe the wellbeing of former elite cricketers.

## 2 Methods

A retrospective cross-sectional questionnaire study was designed for the former cricketers. The study was given favourable opinion by the NHS Health Research Authority (NRES) Committee London Stanmore (REC 15/LO/1274).

There were no known validated questionnaires that included injury, medical, and playing histories for former elite athletes. Therefore, epidemiological questionnaires were developed within the Arthritis Research UK Centre for Sport, Exercise and Osteoarthritis to address these areas in specific sports.^16^ Cricket-specific and wellbeing questions were developed through patient and public involvement (PPI). All invited PPI participants agreed to contribute to PPI discussions, including two physicians and one physiotherapist within elite cricket, and ten former and current cricketers. Six PPI sessions were held, resulting in consensus on the cricket-specific variables and wellbeing measures and the phrasing for capturing these variables, as described in other PPI practice.^17^ Three wellbeing measures stated, “Considering the benefits and risks of my previous participation in cricket, I would do the same again,” “Considering the benefits and risks of my previous participation in cricket, I would recommend this to my children, relatives, or close friends,” and “Did your cricket career enrich your life?” The responses of each wellbeing measure were categorised into “agree”, “undecided”, and “disagree” responses.

All members on the Professional Cricketers’ Association’s (PCA) “former players” contact list were emailed an invitation to participate in the study. One reminder email was sent two weeks after initial contact. Participants could complete the questionnaire online, by telephone or on paper via a postal version. Participant consent and study data were managed using REDCap electronic data capture software hosted at the lead institution.^18^

Wave 1 responses from the English Longitudinal Study of Ageing (ELSA) were used as a cross-sectional normal population sample. ELSA is a longitudinal survey of representative households in England, selecting participants from the Health Survey for England (HSE) aged 50 and above and their young partners to follow with annual surveys.^19^ Wave 1 of ELSA was collected during 2002–2003; core questionnaire data were requested from and provided by the UK Data Service. Demographic data variables were retrieved from Wave 0 of ELSA, when these variables were not collected at Wave 1. Normal population participants aged under 60 were not asked questions regarding joint replacement (TJR).

The self-reported, GP-diagnosed chronic conditions investigated were: heart problems, hypertension, stroke, diabetes, asthma, dementia, OA, THR, TKR, anxiety, and depression. All conditions were posed to the former cricketers in questions that were comparable to the normal population, except for heart problems and joint replacement. For the normal population, heart problems were derived from a positive response to any of six heart conditions including “any other heart trouble”; THR and TKR were derived from positive responses to any hip or knee replacement, respectively. Variable harmonization between the former cricketers and the normal population for each condition can be found in Supplementary material Table A.1. Demographic variables analysed were age, body mass index (BMI), smoking status and ethnicity. The cricket-specific variables of predominant playing position and mean years since retirement were reported for former cricketers. Wellbeing questions were reported for former cricketers and examined their career reflection.

Stata 14 was used for statistical analysis. Prevalences and 95% confidence intervals (CI) of the eleven chronic conditions were calculated for the entire cricketer sample and for the cricketer and normal population samples included in matched analysis.

Standardized morbidity (mortality) ratios (SMRs) were calculated to compare chronic condition prevalences amongst former cricketers to those of the normal, reference population (ELSA). Only male participants were included. Age for both normal and cricketer populations was categorized into 10-year bands, starting with 50–59 years and ending with 80–89 years. BMI for both populations was categorized into WHO classifications for Normal (18.5–25), Overweight (25–30), and Obese (30 + ). WHO subcategories were not used due to small sample sizes in the cricketer sample. The normal population was standardised to the cricketer population by age and BMI using indirect standardization.^20^ Age and BMI were chosen, as they are known risk factors for many of the chronic conditions analysed. Post hoc analysis standardized SMRs by age, BMI and smoking status, due to the association of smoking status with several of the conditions investigated.

## 3 Results

**Fig. 1:**
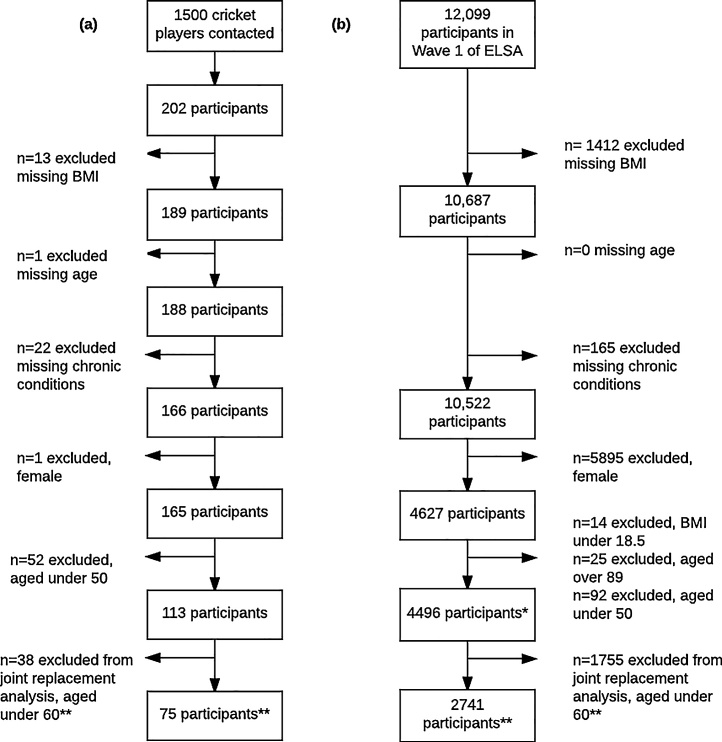
(a) Former cricketers included in analysis, from recruitment to final sample size; (b) normal population (ELSA Wave 1 participants) included in analysis. *Normal population participants aged 50 and over, and matched for age and BMI with former cricketers. **Normal population participants aged under 60 were not asked joint replacement questions (THR, TKR); therefore, participants aged 60 and over in both population samples were matched for THR and TKR.

Recruitment for the former cricketer study lasted four months, contacted 1500 former cricketers and resulted in a response rate of 13%. Of cricketers that requested to participate, 80.2% submitted a completed questionnaire. Fig. 1 shows how the samples from the former cricketer and normal populations were determined for analysis. Complete responses to age, BMI, and all chronic conditions analysed were required for inclusion in prevalence calculations. For age- and BMI-adjusted SMRs, only male participants aged 50–89 years with a BMI over 18.5 were included. Due to the normal population’s questionnaire, only participants aged 60 and over in both populations were included in THR and TKR SMR analysis.

Table 1 shows participant characteristics for the samples included in analyses. The former cricketers excluded from SMRs due to age (n = 52) were not significantly different to those included in SMRs (n = 113) for any characteristic but age (p > 0.05). The normal population (n = 4496) was significantly different from the former cricketers included in SMR analysis (n = 113) only for smoking status (p < 0.001).

**Table 1:** Participant characteristics of entire former cricketer sample, and of former cricketer and normal (ELSA) populations included in matched analysis.

Characteristic	All cricketers (n = 165)	Cricketers aged 50+ (n = 113)	ELSA (n = 4496)
Age (years)			
Mean (SD)	57.2 (14.2)	65.1 (9.1)	64.2 (9.6)
Range	28–88	50–88	50–89

Body mass index			
Mean (SD)	27.6 (3.6)	27.9 (4.0)	27.6 (3.8)
Range	21.6–53.1	21.6–53.1	21.4–53.1

Smoking status*			
Current smoker	9 (5.5%)	6 (5.4%)	775 (17.4%)
Does not smoke	137 (83.5%)	92 (82.1%)	1170 (26.3%)
Ex-smoker	18 (11.0%)	14 (12.5%)	2510 (56.3%)

Ethnicity			
White	156 (95.7%)	107 (96.4%)	4358 (96.9%)
Black, Asian, or mixed	7 (4.3%)	4 (3.6%)	138 (3.1%)

Playing position			
Batsman	43 (26.1%)	34 (30.1%)	–
Wicketkeeper	18 (10.9%)	11 (9.7%)	–
Fast bowler	78 (47.3%)	51 (45.1%)	–
Spin bowler	26 (15.7%)	17 (15.1%)	–

Years since retirement from cricket [mean (SD)]	24.9 (13.7)	30.6 (11.4)	–

* Significant difference between former cricketer and normal populations included in matched analysis (p < 0.001).

Table 2 shows the prevalences and 95% CI of the chronic conditions analysed. Table 2 also shows the age- and BMI-adjusted SMRs and 95% CI for the chronic conditions analysed. An SMR and 95% CI less than 1.0 indicates a statistically significantly lower prevalence in the former cricketers, while an SMR and 95% CI greater than 1.0 indicates a statistically significantly higher prevalence in the former cricketers compared to the normal population.

**Table 2:** Prevalence (%) and 95% confidence interval (CI) for each chronic condition in the entire former cricketer sample and in the former cricketers and normal (ELSA) population included in matched analysis, and the age- and BMI-adjusted SMRs and 95% CI for chronic conditions in the former cricketer population compared to the normal population (ELSA), with statistically significant results in bold.

Outcome	All cricketers (N = 165)	Cricketers aged 50+ (N = 113)	ELSA (N = 4496)	SMR analysis
	% Overall (95% CI)	% Overall (95% CI)	% Overall (95% CI)	Age- and BMI-adjusted SMR (95% CI)
Heart Problems	9.7% (6.0–15.3%)	13.3% (8.1–21.0%)	23.1% (21.9–24.4%)	**0.55 (0.33–0.91)**
Hypertension	23.6% (17.7–30.8%)	31.9% (23.8–41.1%)	36.4% (35.0–37.8%)	0.84 (0.60–1.16)
Stroke	1.2% (0.3–4.8%)	1.8% (0.4–6.9%)	4.5% (3.9–5.1%)	0.38 (0.09–1.52)
Diabetes	4.2% (2.0–8.7%)	6.2% (2.9–12.6%)	8.9% (8.1–9.8%)	0.65 (0.31–1.35)
Asthma	15.8% (10.9–22.2%)	15.0% (9.5–23.0%)	10.2% (9.3–11.1%)	1.47 (0.91–2.37)
Dementia^b^	0	0	0.6% (0.4–0.9%)	
Osteoarthritis	44.2% (36.8–52.0%)	51.3% (42.0–60.5%)	13.4% (12.4–14.4%)	**3.64 (2.81–4.71)**
Total Hip Replacement	7.3% (4.2–12.4%)	14.7% (8.2–24.9%)	3.6% (3.0–4.4%)	**3.99**^a^**(2.21–7.20)**
Total Knee Replacement	5.5% (2.8–10.2%)	10.7% (5.3–20.2%)	2.5% (2.0–3.2%)	**3.84**^a^**(1.92–7.68)**
Anxiety	10.3% (6.5–16.0%)	12.4% (7.4–20.0%)	3.4% (2.9–3.9%)	**3.95 (2.34–6.67)**
Depression	6.7% (3.7–11.7%)	8.8% (4.8–15.8%)	4.2% (3.7–4.9%)	**2.22 (1.20–4.14)**

a ELSA data only has complete data for those aged 50 and over, and only has THR and TKR data for those aged 60 and over. The cricketer population for each of these outcomes has been matched accordingly, with 75 cricketers and 2741 ELSA participants in the THR and TKR outcome analysis.

b No cricketers reported dementia.

Two former cricketers from the matched analysis were excluded from wellbeing analysis due to missing data (total valid n = 111). Of those asked, 97.3% (n = 108) of cricketers agreed they would undertake their cricket career again; the remaining 2.7% (n = 3) were undecided. 93.7% (n = 104) of cricketers agreed they would recommend their cricket career to a loved one; 4.5% (n = 5) were undecided. Finally, 98.2% (n = 109) of cricketers agreed their cricket career enriched their lives; the remaining 1.8% (n = 2) disagreed.

Due to the association of smoking status with several of the conditions investigated and the significant difference in smoking status between the former cricketers and normal population, a post hoc analysis standardized the SMRs by age, BMI, and smoking status. The effect size of some SMRs changed in post hoc analysis; however, including smoking status did not significantly change any SMRs.

## 4 Discussion

To the best of our knowledge, this research is the first comprehensive assessment of the long-term health of former cricketers. Cardiac conditions were lower in former cricketers, while musculoskeletal and mental health conditions were significantly higher compared to the normal population. The majority of former cricketers reflected positively on their cricket careers. These results identify mental and musculoskeletal health as targets for resource provision during and after players’ cricket careers. These findings also suggest that most cricketers see the benefits outweighing the risks in cricket, as most cricketers agreed they would “do the same again”.

The prevalence of heart problems was significantly lower in the former cricketers than the normal population, with an SMR of 0.55, suggesting a protective effect for the former cricketers. This is consistent with existing literature reporting decreased rates of CHD and other cardiovascular outcomes with increased physical activity.^6^, ^21^, ^22^ Even when adjusting for smoking status, which was significantly different between the populations, this finding was maintained.

The SMRs for hypertension and diabetes suggested a trend toward a lower prevalence in former cricketers compared to the normal population, though this trend was not statistically significant. Such a trend is consistent with current literature, as hypertension and diabetes are often associated with cardiovascular conditions and beneficially influenced by physical activity.^6^, ^8^ There was no significant difference between the former cricketers and normal population in the prevalence of stroke, though stroke would have been expected to see the same positive impact of physical activity as heart problems. Stroke was rare in both populations, resulting in wide confidence intervals and making a statistically significant difference unlikely. No former cricketers in this study reported dementia, perhaps due to data collection bias as the study was primarily advertised by email. The effect size did not change significantly for any of these conditions when adjusting for smoking status. However, the confidence intervals narrowed following the trend expected, given the physical activity and smoking habits of the former cricketers.

OA prevalence was higher in the former cricketers than in the normal population, consistent with current literature.^4^, ^11^, ^12^ Elite athletes can see high rates of injury with the intensity of their training and competition. Important to note, though, is that injury rates depend on the sport. While elite football players have injury incidence rates of 77.3 injuries per 1000 player-hours, elite cricketers have injury rates ranging from 1.39 to 4.87 injuries per 1000 player-hours.^23^, ^24^ Joint injury rates may contribute to the higher rates of OA and TJR found in former cricketers and other former elite athletes.^4^, ^12^ A more definitive measure of OA can be TJR, which is most often a treatment for advanced OA. The higher rate of TJR in the former cricketers than the normal population may indicate more advanced OA in the former cricketers and supports the increased OA finding as a true result. There are, however, a few factors relevant to this population of former cricketers that may have affected the higher reported rate of OA and TJR. The authors are aware that the phrase “wear and tear” is commonly used by medical professionals that work with cricketers, and is not always meant as a diagnosis of OA. These elite athletes may be more likely to receive an OA diagnosis or treatment with a TJR because they are more likely to have access to and be seen by a doctor, and may be more likely to seek treatment in order to maintain activity. These factors may have overestimated the prevalence of OA and TJR in the former cricketers.

Rates of anxiety and depression were significantly higher in the former cricketers than the normal population. More recent reports for the UK estimate anxiety prevalence at 6.6%, higher than this normal population’s prevalence of 3.4%, and estimate depression at 3.8%, lower than this normal population’s prevalence of 4.2%.^25^ A recent study of current and former professional South African cricketers found 4-week prevalence of symptoms of anxiety and depression to be 37% in current cricketers and 24.3% in former cricketers, higher than the prevalences reported here.^26^ Importantly, the symptoms of anxiety and depression were assessed in the South African cricketers, which may be more sensitive than this study’s measure of lifetime clinical diagnoses. Still, a diagnosis captured in this study may have been received after retirement from elite sport, a transition period many elite athletes find difficult.^15^ This raises the importance of future research asking both if participants have “ever had” a mental health diagnosis, and if they “currently have” a diagnosis. The PCA has done a great deal of work in the last 5 years to raise awareness of and foster openness about mental health in former cricketers. The reported prevalences of anxiety and depression in the former cricketers may indicate that this work has encouraged more former cricketers to seek help and receive a diagnosis, compared to the normal population. Previous research has also found that injury history, pain and OA play a role in anxiety and depression rates for elite athletes; this may warrant further analysis within former cricketers.^13^, ^27^

Of further note are the wellbeing measures in this study, which support the model of separating mental health into two distinct categories of mental illness and mental health, or wellbeing.^28^ Keyes propose that the presence of mental illness, such as anxiety or depression, does not “imply the absence of mental health”.^28^, ^29^ While diagnosed anxiety and depression were higher than the normal population, the vast majority of former cricketers reflected positively on their cricket career. Over 98% of former cricketers felt that cricket “enriched” their lives and no former cricketers felt, upon reflection of the benefits and risks of their cricket career, that they would not do the same again. Additionally, over 93% of former cricketers agreed that they would recommend their career in cricket to their loved ones. This reflects the players’ outweighing of benefits over the risks in cricket, even at the elite level.

This study had a few strengths and limitations of note. While elite cricket participation requires intense physical activity, this study assumed the health benefits of this activity were maintained after retirement from elite cricket, without accounting for current activity levels. This is the largest cohort of former elite cricketers for the study of long-term health and it is possible that the 13% response rate limits generalizability to all cricketers. Although these cricketers’ age distribution was representative of the PCA’s entire former player population, we cannot exclude selection bias. Former cricketers with health problems may be more likely to complete the questionnaire, leading to an overestimation of the prevalence and risk of conditions in the former cricketers. However, the decreased risk of heart problems argues against this as a major bias.

Using ELSA as the normal population required exclusion of former cricketers aged under 50. The exclusion of younger cricketers may have decreased the power of some results, like hypertension. The former cricketer and normal populations were significantly different in smoking status, though post hoc analysis did not show smoking status to significantly change any SMRs.

The former cricketer population excluded the only female participant. The number of women that have played cricket at higher than a recreational level in the UK is very low. However, the wider recruitment of former female cricketers would provide a fuller picture of the long-term impacts of cricket.

Finally, variation in question phrasing posed to the former cricketers versus the normal population may have created bias in reporting. Table A.1 in Supplementary material shows the comparison of question phrasing. It is noteworthy that for OA, only a precise self-reported diagnosis of “osteoarthritis” would have defined a case for the normal population. This type of “strict” definition has been shown to have a lower sensitivity than one that includes phrases that patients might use to describe their diagnosis.^30^ For anxiety and depression, the normal population had to first respond positively to “Any emotional, nervous, or psychiatric problems”; the former cricketers were presented directly with the conditions “anxiety” and “depression.” The definition of OA and the extra layer of questioning for anxiety and depression for the normal population may have underestimated their prevalence of these conditions.

## 5 Conclusions

This study presented the largest cross-sectional cohort of former elite cricketers to investigate long-term health. This population of former elite cricketers had significantly lower rates of heart problems and non-significant but lower trending rates of hypertension and diabetes than the normal population sample. These former cricketers also had higher rates of OA, THR, TKR, anxiety and depression than the normal population. Over 97% of former cricketers agreed with the statement that they would “do the same again” with regards to their cricket career. Understanding the long-term health of former elite cricketers can inform risk prevention strategies in current players of this popular sport and inform resource provision for players transitioning out of professional cricket. Future studies should explore mechanisms and risk factors for the higher rates of musculoskeletal conditions.

## Practical implications

• Heart problems were less prevalent in former elite cricketers than a normal population, emphasizing the benefits of physical activity.
• Osteoarthritis and total hip and knee replacement, as well as anxiety and depression were more prevalent in former elite cricketers than a normal population.
• Higher rates of joint and mental health conditions in former elite cricketers provide target areas for the sport to prevent and mitigate these conditions for future players.
• Understanding the possible positive and negative outcomes of long-term sport participation contributes to informed participation.
