# Supplementary material for: Osteoarthritis and other long-term health conditions in former elite cricketers
Source: J Sci Med Sport. 2018 Jun;21(6):558–63. doi: 10.1016/j.jsams.2017.10.013 (PMC5964309; doi:10.1016/j.jsams.2017.10.013)
Supplement: Supplementary file 1 [file mmc1.docx]

**Supplementary Material**

Table A.1. Variable harmonization between the former cricketers and the normal population (ELSA) for each outcome. Answer options for the former cricketers were “yes”, “no” and “don’t know” for each outcome. Branching questions are indicated with instructions in italics.

| **Outcome** | **Question(s), answer(s) posed to former cricketers** | **Question(s), answer(s) posed to normal population (ELSA)** | **Harmonised Variable** |
| --- | --- | --- | --- |
| *Introductory question:* | Have you ever been told you have any of the following by a Doctor? | Has a doctor ever told you that you [have/have had] any of the conditions on this card? What others? |  |
| Asthma | - Asthma | - Asthma | - Asthma |
| Dementia | - Dementia | - Dementia, organic brain syndrome, senility, or any other serious memory impairment | - Dementia |
| Diabetes | - Diabetes | - Diabetes or high blood sugar | - Diabetes |
| High blood pressure | - High blood pressure | - High blood pressure or hypertension | - High blood pressure |
| Stroke | - Stroke | - A stroke (cerebral vascular disease) | - Stroke |
| Heart problems | - Heart problems | - Angina - A heart attack (including myocardial infarction or coronary thrombosis) - Congestive heart failure - A heart murmur - An abnormal heart rhythm - Any other heart trouble | - Heart problems: positive response to any of the six heart conditions posed to the normal population |
| Anxiety Depression | - Anxiety - Depression | *If answered,* “Any emotional, nervous, or psychiatric problems”  *Then asked,* “What type of emotional, nervous, or psychiatric problems do/did you have?” |  |
|  |  | - Anxiety - Depression | - Anxiety - Depression |
| Osteoarthritis | Have you ever been told that you have wear and tear, degeneration, or osteoarthritis by a doctor? | *If answered,* “Arthritis (including osteoarthritis, or rheumatism)” *then asked,* “Which type or types of arthritis do you have?” |  |
|  |  | - Osteoarthritis | - Osteoarthritis |
| Hip and Knee replacement | “Have you ever had joint replacement surgery?” *If answered,* “Yes” *then asked,* “If yes, where?” | “Have you ever had any joint replacements?” *If answered,* “Yes” *then asked,* “Which joints did you have replaced?” | - THR: positive response to one or both hips, or hip(s) and knee(s) |
|  | - Hip - Knee | - Hip - Both hips - Knee - Both knees - Hip(s) and knee(s) | - TKR: positive response to one or both knees, or hip(s) and knee(s) |
